# Facilitation of Auditory Comprehension After Theta Burst Stimulation of Wernicke's Area in Stroke Patients: A Pilot Study

**DOI:** 10.3389/fneur.2019.01319

**Published:** 2020-01-08

**Authors:** Viviana Versace, Kerstin Schwenker, Patrick B. Langthaler, Stefan Golaszewski, Luca Sebastianelli, Francesco Brigo, Elke Pucks-Faes, Leopold Saltuari, Raffaele Nardone

**Affiliations:** ^1^Department of Neurorehabilitation, Hopsital of Vipiteno-Sterzing, Vipiteno-Sterzing, Italy; ^2^Research Unit for Neurorehabilitation of South Tyrol, Bolzano, Italy; ^3^Department of Neurology, Christian Doppler Klinik, Paracelsus Medical University, Salzburg, Austria; ^4^Karl Landsteiner Institut für Neurorehabilitation und Raumfahrtneurologie, Salzburg, Austria; ^5^Department of Neurology, Franz Tappeiner Hospital, Merano, Italy; ^6^Department of Neuroscience, Biomedicine and Movement Science, University of Verona, Verona, Italy; ^7^Department of Neurology, Hochzirl Hospital, Zirl, Austria

**Keywords:** repetitive transcranial magnetic stimulation, theta burst stimulation, fluent aphasia, Wernicke's area, auditory comprehension

## Abstract

**Introduction:** Single-pulse transcranial magnetic stimulation (TMS) and high-frequency repetitive TMS (rTMS) over Wernicke's area were found to facilitate language functions in right-handed healthy subjects. We aimed at investigating the effects of excitatory rTMS, given as intermittent theta burst stimulation (iTBS) over left Wernicke's area, on auditory comprehension in patients suffering from fluent aphasia after stroke of the left temporal lobe.

**Methods:** We studied 13 patients with chronic fluent aphasia after an ischemic stroke involving Wernicke's area. iTBS was applied in random order to Wernicke's area, the right-hemisphere homologous of Wernicke's area, and the primary visual cortex. Auditory comprehension was blind assessed using the Token test before (T0), 5 (T1), and 40 min (T2) after a single session of iTBS.

**Results:** At the first evaluation (T1) after iTBS on left Wernike's area, but not on the contralateral homologous area nor on the primary visual cortex, the scores on the Token test were significantly increased. No significant effects were observed at T2.

**Conclusion:** We demonstrated that a single session of excitatory iTBS over Wernicke's area was safe and led to a transient facilitation of auditory comprehension in chronic stroke patients with lesions in the same area. Further studies are needed to establish whether TBS-induced modulation can be enhanced and transformed into longer-lasting effects by means of repeated TBS sessions and by combining TBS with speech and language therapy.

## Introduction

Repetitive transcranial magnetic stimulation (rTMS) seems to be particularly effective in promoting cortical plasticity in stroke (1, 2). High-frequency rTMS over the lesioned motor cortex increased motor-evoked potential (MEP) amplitude (3). Theta burst stimulation (TBS) is an rTMS stimulation protocol which presents several advantages: use of low intensities, robust, and long-lasting effects both in normal subjects (4, 5) and in chronic stroke patients (6, 7), and very short duration of a single session. Different patterns of TBS delivery (continuous vs. intermittent) produce opposite effects on synaptic efficiency of the stimulated motor cortex (4–8). The paradigm known as intermittent TBS (iTBS) produces a consistent long-term potentiation (LTP)-like effect, causing a prolonged increase of motor cortex excitability (4).

The possibility of boosting cortical synaptic plasticity through non-invasive stimulation generates important implications in rehabilitative strategies of stroke patients.

Aphasia is one of the most common poststroke cognitive disorders. Different stroke-damaged neural pathways produce different language deficits. Lesions causing impairment of fluency are typically located in the left inferior frontal gyrus, including Broca's area. Deficits in the auditory comprehension depend on injury of Wernicke's area, traditionally located in the posterior section of the left superior and middle temporal gyri (9–11).

Several studies indicated that inhibitory rTMS over the contralesional inferior frontal gyrus improves recovery from poststroke non-fluent aphasia by reducing right hemisphere hyperactivity and transcallosal inhibition on left Broca's area [for a review: (12, 13)]. With small left hemisphere lesions, perilesional regions are recruited to subserve the recovery of language function. Few studies targeted the ipsilesional frontotemporal regions in aphasic patients. Cotelli et al. observed an improvement in object-naming following 20 Hz rTMS over the left dorsolateral prefrontal cortex in three chronic stroke patients (14). Functional MRI-guided, excitatory rTMS applied to the affected Broca's area improved language skills in patients with chronic poststroke aphasia (15). Ten Hertz rTMS over to the lesional inferior frontal gyrus improved repetition and naming tasks in a chronic stroke patient with non-fluent aphasia (16). More recently, iTBS was applied to the fMRI determined residual left frontotemporal language-responsive regions in chronic stroke patients suffering from different types of aphasia; an improvement in language performances correlated with increased activation of the stimulated regions (17, 18).

In right-handed healthy subjects, single pulse or repetitive TMS over Wernicke's area produced a facilitation in picture-naming tasks (19, 20).

In the present study, we aimed at evaluating the effects of excitatory iTBS over left Wernicke's area on auditory comprehension in patients with poststroke fluent aphasia and in healthy controls.

## Materials and Methods

### Patients

Thirteen right-handed patients (mean age, 68.2; range, 54–78 years) were enrolled in the study, all of whom suffered from fluent aphasia after first event left middle cerebral artery stroke of different etiologies involving the posterior perisylvian region. Mean time since stroke was 5.3, range of 2–10 years.

Participants were screened using the Boston Diagnostic Aphasia Examination (21). Auditory comprehension was assessed by a 36-item version (shortened form) of the Token test (22). The total score ranged from 0 (worst performance) to 36 (best performance).

**Figure 1:**
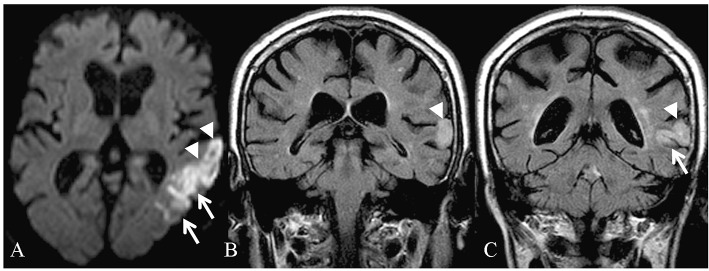
Magnetic resonance imaging of the patient 1 in the acute phase: restricted diffusion is identifiable in the left temporal lobe **(A)** on diffusion-weighted imaging (DWI). Coronal fluid-attenuated inversion recovery (FLAIR) images **(B,C)** demonstrate increased signal in the region of the superior temporal gyrus, on the left.

The clinical and demographic characteristics of the patients are illustrated in Table 1. The magnetic resonance imaging (MRI) findings of the patient 1 are shown in Figure 1.

**Table 1:** Demographic characteristics of patients and scoring on the Boston Diagnostic Aphasia Examination.

					**Boston diagnostic aphasia examination**					
**Patients**	**A (y) G**	**Time (y) since stroke**	**Auditory comprehension**		**Fluency**		**Repetition**		**Naming**	
			**Word**	**Command**	**Articulation**	**Phrase length**	**Words**	**Sentences**	**Animal**	**Body part**
1	54 M	2	27/100	7/10	7/7	7/7	9/10	7/10	3/10	3/10
2	68 M	4	20	4	7	6	1	0	1	0
3	64 F	2	24	6	7	7	2	1	2	2
4	72 M	5	15	3	6	6	0	0	1	0
5	56 M	4	13	3	7	6	0	0	1	2
6	73 M	8	17	5	7	7	1	0	2	1
7	66 F	7	40	8	7	7	3	1	4	4
8	75 M	6	34	7	7	7	3	1	3	1
9	78 F	10	16	3	7	6	1	0	2	1
10	69 F	6	19	4	6	6	1	0	2	1
11	72 M	4	22	5	7	7	1	0	1	0
12	75 F	8	17	3	7	6	1	1	1	1
13	65 M	3	35	8	7	7	3	2	3	3

*A, age; G, gender; y, years*.

All patients showed auditory comprehension deficits but were able to give informed consent for the research study.

Exclusion criteria were contraindication to TMS and severe medical problems, such as heart failure or respiratory diseases, a history of cerebrovascular disease, or other neurological or psychiatric diseases.

All patients provided informed consent before participation in the study, which was performed according to the declaration of Helsinki and approved by the Ethics Committee.

### Transcranial Magnetic Stimulation

Magnetic stimulation was performed using a high-power Magstim 200 magnetic stimulator (The Magstim Company Ltd., Whitland, UK), connected with a figure-of-eight coil with external loop diameters of 9 cm. It was held over the left motor cortex at the optimum scalp position to elicit MEPs in the contralateral first dorsal interosseous muscle. The induced current flowed in a posteroanterior direction. Active motor threshold (AMT) was defined as the minimum stimulus intensity that produced a liminal MEP (~200 μV in 50% of 10 trials) during isometric contraction of the tested muscle (23). The iTBS protocol consisted of 10 bursts, each of which was composed of three stimuli at 50 Hz, repeated at a theta frequency of 5 Hz every 10 s for a total of 600 stimuli (total duration, 200 s) (4). iTBS was delivered in random order over Wernicke's area, the homologous temporal area of the right hemisphere, and to the primary visual cortex using a high frequency magnetic stimulator (Magstim Rapid, The Magstim Company Ltd., Whitland, UK) connected to a standard Magstim figure-of-eight coil. The coil was positioned tangentially to the skull, with the handle parallel to the sagittal axis and pointing occipitally. For the stimulation of Wernicke's area (W1) and of the right-hemisphere homologous area (W2), the coil was centered over CP5 and CP6 of the International 10–20 System, respectively. According to the literature, this site correlates best with the location of Wernicke's area (24–26). The primary visual cortex was stimulated at the occiput (27). The stimulation intensity was defined in relation to AMT; an intensity of 80% AMT was used.

For each stimulation site, the Token test was administered at baseline (T0), 5 min after iTBS (T1), and 40 min after iTBS (T2). To evaluate the specificity of the TBS effect, the patient were given iTBS on different cortical regions on separate days. The order of the iTBS treatment was randomly assigned.

### Statistical Analysis

For statistical analysis, we used the software R [R Core Team (28)], more precisely the package nparLD (29), which was specifically designed for the non-parametric analysis of longitudinal data. We decided to use a non-parametric method since the results of the Token test are count data with 37 possible outcomes and therefore cannot possibly be normally distributed. In combination with a sample size of 13 participants, this makes classical methods such as repeated measures ANOVA unreliable. nparLD provides several test statistics. We used the ANOVA-type test statistic, since it tends to perform better for small sample sizes (29).

As a measure of effect, we used the relative treatment effect (RTE) provided by nparLD. The RTE can be interpreted in the following way.

For comparisons between two groups, A and B, the RTE for A describes the probability that a randomly drawn subject from group A scores higher on the outcome variable than a randomly drawn subject from group B, plus half the probability that a randomly drawn subject from group A scores the exact same score on the outcome variable as a randomly drawn subject from group B. The RTE thus lies between 0 and 1, with 0.5 meaning no effect and 0 and 1 meaning complete separation of the two groups. For comparisons with more than two groups the RTE for a group is the probability that a random subject drawn from this group scores higher than a random subject drawn from the entire sample, plus half the probability that a random subject drawn from this group scores exactly the same on the outcome variable than a subject randomly drawn from the entire sample. In this case, the RTE lies between 1/(2*N*) and 1 – 1/(2*N*), where *N* is the number of groups.

Our testing strategy was as follows: We modeled time and area as repeated factors and performed a global test for an area effect, a time effect, and an interaction effect on the Token test score, each at alpha/3. If there was no significant interaction and a significant area effect, we performed a pairwise follow-up test comparing all areas, pooling all time points together. If there was no significant interaction effect and a significant time effect, we performed a pairwise follow-up test comparing all time points, pooling all areas together. If there was a significant interaction effect, we first tested for a time effect within each area and an area effect within each time point at alpha/9, respectively. If any of these was significant, we performed the corresponding pairwise follow-up test, comparing all time points within that area or all areas within that time points, respectively. Owing to the closed testing principle (30), it suffices to perform these follow-up tests at alpha/9. We chose alpha to be 0.05. All *p*-values given in *Results* have been corrected for multiple testing, that is, multiplied by either 3 or 9, and can thus be interpreted as significant if they are below 0.05. Figures were created using the R package ggplot (31).

## Results

**Figure 2:**
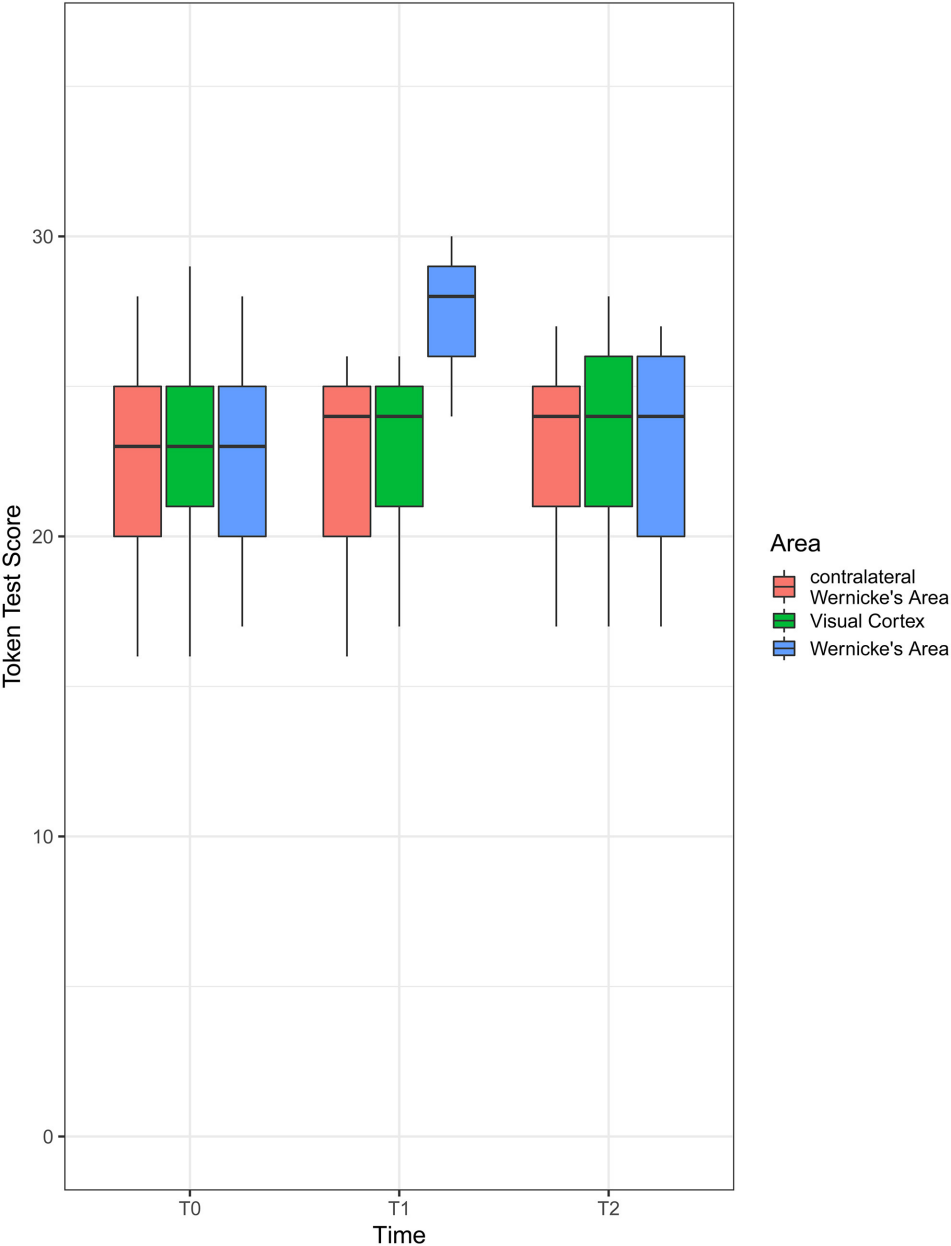
Boxplot of Token test scores by area and time. Shown is the first quartile (bottom of the box), median (thick line in the middle of the box), and third quartile (top of the box). The whiskers extend to the furthest observation from the box that is still within 1.5 × interquartile range (IQR) (the interquartile range, i.e., value of third quartile – value of first quartile). Observations more than 1.5 × IQR away from the box are shown as individual points (not present here).

There was a significant area [*F*_(1.68, ∞)_ = 31.29; *p* < 0.001], time [*F*_(1.69, ∞)_ = 12.10; *p* < 0.001], and interaction [*F*_(1.74, ∞)_ = 44.67; *p* < 0.001] effect on the Token test score. The RTEs are provided in Table 2. An illustration is given in Figure 2.

**Table 2:** Table of relative treatment effects (RTEs) for every time × area combination and overall time/area level.

**Time/area**	**Wernicke's area**	**Contralateral Wernicke's area**	**Visual cortex**	**Overall**
T0	0.45	0.46	0.46	0.46
T1	0.85	0.4	0.47	0.57
T2	0.47	0.52	0.48	0.5
Overall	0.59	0.44	0.47	

There was a significant time effect in Wernicke's area [*F*_(1.16, ∞)_ = 72.62; *p* < 0.001; RTEs: T0 = 0.36, T1 = 0.76, T2 = 0.38] but not in the contralateral Wernicke's area [*F*_(1.73, ∞)_ = 1.80; *p* = 1; RTEs: T0 = 0.52, T1 = 0.46, T2 = 0.51] or the visual cortex [*F*_(1.54, ∞)_ = 0.56; *p* = 1; RTEs: T0 = 0.48, T1 = 0.50, T2 = 0.51].

There was a significant area effect at T1 [*F*_(1.13, ∞)_ = 80.30; *p* < 0.001; RTEs: Wernicke = 0.79, contralateral Wernicke = 0.33, visual cortex = 0.38], but not at T0 [*F*_(1.68, ∞)_ = 0.14; *p* = 1; RTEs: Wernicke = 0.49, contralateral Wernicke = 0.50, visual cortex = 0.50] or T2 [*F*_(1.63, ∞)_ = 0.48; *p* = 1; RTEs: Wernicke = 0.50, contralateral Wernicke = 0.49, visual cortex = 0.52].

For Wernicke's area, there was a significant difference between T0 and T1 [*F*_(1, ∞)_ = 79.92; *p* < .001; RTEs: T0 = 0.30, T1 = 0.70], as well as between T1 and T2 [*F*_(1, ∞)_ = 70.33; *p* < 0.001; RTEs: T1 = 0.70, T2 = 0.30] but no significant difference between T0 and T2 [*F*_(1, ∞)_ = 2.56; *p* = 1; RTEs: T0 = 0.49, T1 = 0.51].

For T1, there was a significant difference between Wernicke's area and contralateral Wernicke's area [*F*_(1, ∞)_ = 89.72; *p* < 0.001; RTEs: Wernicke = 0.72, contralateral Wernicke = 0.28] as well as between Wernicke's area and the visual cortex [*F*_(1, ∞)_ = 89.25; *p* < 0.001; RTEs: Wernicke = 0.71, visual cortex = 0.29]. There was also a significant difference between contralateral Wernicke's area and the visual cortex [*F*_(1, ∞)_ = 9.39; *p* = 0.02; RTEs: contralateral Wernicke = 0.46, visual cortex = 0.54].

Overall, we can state that the time profile of the Token test score differed depending on the area that iTBS stimulation was applied to. While there is no evidence to conclude that the time profiles for contralateral Wernicke's area of the visual cortex are not flat, there is strong evidence that the profile is not flat when stimulation is applied to Wernicke's area. At T0 and T2, there is no evidence for a difference between groups but strong evidence of difference at T1. Here, all pairwise comparisons were different, albeit the difference between contralateral Wernicke's area and the visual cortex was small.

## Discussion

In this pilot study, we highlight the transient facilitatory effect of a single session of iTBS over Wernicke's area on a simple auditory comprehension task (the Token test) in 13 right-handed chronic stroke patients with fluent aphasia. The results were highly specific for stimulation of lesional Wernicke's area compared to the right-hemisphere homologous region and to the primary visual cortex. A significant facilitation of speech comprehension was detected at T1, 5 min after iTBS over Wernicke's area. This effect subsequently decreased, and after 40 min, facilitation of auditory comprehension was no longer detectable.

We were able to exclude the possibility that iTBS may have functioned as a warning stimulus, raising attention and thereby resulting in a shorter reaction time, by demonstrating that the noise produced by iTBS on the “control” cortical regions had no impact on auditory comprehension. We also excluded the possibility of an order effect by varying the sequence of iTBS treatments.

In animal models, the repeated electrical stimulation of brain neural circuits changes the synaptic strength, modulates postsynaptic Ca^2+^ influx and leads to synaptic LTP or long-term depression (LTD). The persistent LTP/LTD-dependent changes in synaptic efficacy are the basis of learning and memory processes, as well as the acquisition or recovery of sensorimotor functions (32–35). Evidence from the past 20 years suggests that rTMS can potentially cause similar changes in the cerebral human cortex (36, 37).

A single session of iTBS (600 pulses) over the primary motor cortex resulted in increased cortical excitability indexed by MEP amplitude for ~30 min following stimulation (4). Di Lazzaro et al., provided direct demonstration of post-iTBS enhancement of the later I waves of descending corticospinal volleys evoked by TMS of the motor cortex by direct epidural recording in conscious patients (38). The facilitatory after-effects effect of iTBS rely on *N*-methyl-d-aspartate receptors, thus reflecting LTP-like plasticity mechanisms (39–41).

Previous findings demonstrate that the excitability of the impaired motor cortex in acute stroke can be effectively enhanced by iTBS (6, 7). As LTP-like plasticity can occur in all major cortices, it can be hypothesized that an iTBS-induced facilitation might also occur in temporal and other cortical areas.

In the immediate vicinity of an acute lesion in the motor areas, the cortical excitability increases, possibly due to the cancellation of GABAergic lateral inhibition (42). Hyperexcitability and facilitation of theta burst induced LTP in the surround of a cortical infarct were demonstrated in animal models in the acute poststroke phase and concurred with activity-dependent plasticity and functional recovery (43–46). The stronger activation in intact motor and language areas found in functional imaging could be explained by these mechanisms (47). The improvement of sensorimotor neurological deficits in the first few weeks or months after a stroke likely relies on hyperactivation of spared lesional–perilesional neurons, which facilitates long-term plasticity, fiber sprouting, and synaptogenesis. In the chronic stage, functional recovery depends more on recruiting of existing but functionally silent synaptic connections located close to the compromised area or of alternative neural routes that are anatomically remote but functionally related (48, 49).

In addition, the recovery of language abilities after a unilateral brain lesion classically depends on two possible functional mechanisms: (1) a laterality shift, with activation of homotopic right-hemispheric areas when the tissue damage of the left-hemisphere language network regions is very large and concurs with permanent impairment and (2) recovery of perilesional areas of the left hemisphere with reactivation of left hemisphere network (50–52).

A growing body of evidence highlights the crucial role of the left hemispheric spared areas in aphasia recovery also at chronic stages (53–58). Functional neuroimaging studies also confirmed that improved speech and language functioning following aphasia treatment relies, at least partly, on the activation of spared regions of the left hemisphere (59–62). Similarly to what happens in the motor cortex, also in the left hemispheric language network, neural pathways that in healthy brain play a minor function or are even silent, might become “unmasked” or disinhibited after a partial damage of the system, and replace primary connections (50, 63).

Based on these considerations, it is reasonable to assume that activating preserved neural pathways in left temporal lobe by means of non-invasive brain stimulation, in combination with conventional speech therapy, may help the rehabilitation of poststroke aphasia. A few preliminary TMS and transcranial direct current stimulation approaches targeting the left temporal region in chronic aphasic patients showed indeed significant improvement in language abilities (18, 64–66). An alternative promising approach to foster audiomotor integration ability, tested so far only in patients with disorder of consciousness due to severe brain injury, consisted in pairing auditory with transcranial magnetic stimuli on the primary motor area, using the stimulation paradigm known as paired associative stimulation, which induces associative LTP or LTD-like neuronal plasticity (67).

The present findings provide evidence of safety, feasibility, and efficacy of iTBS delivered over lesional Wernicke's area in chronic stroke patients with fluent aphasia. The effects were, however, short lived. Repeated sessions of iTBS might increase the magnitude and duration of the beneficial effects and transform them into clinically relevant changes possibly combined with speech and language therapy.

Moreover, considering the outcome to a single session of iTBS on Wernicke's area as here proposed, evaluating aphasic patients even at an earlier stage could represent a predictive marker of brain stimulation responsiveness and allow a reasonable allocation in further rehabilitative protocols.

The findings of this study must be seen in light of some limitations. First, we did not confirm the site of stimulation by the use of other methods such as MRI. The possibility indeed remains that the observed effect might be due to inadvertent activation of other cortical areas such as the inferior parietal cortex or the sensory cortex. On the other hand, Wernicke's area's precise location is still under debate, whether within the posterior temporal or inferior parietal regions (68–70), and is often defined on a functional basis (71, 72). In the present study, the improvement of language comprehension subsequent to iTBS was specifically observed for the “Wernicke's area” target and not for the other targets indicating a specific modulation of this cortical region. In any case, even if stereotaxic placement of the coil provides best accuracy, stereotaxic approaches are expensive and not always available. The use of the 10–20 system for TMS positioning is easily applicable and low cost and may reach desired cortex regions on a larger scale level (73). Wernicke's area was previously targeted basing the coil positioning on the 10–20 system (19, 20, 74).

The second limitation concerns the outcome measure. Assessing language function with a single test due to limited evaluation time and patient compliance precludes a deeper investigation of the clinical improvement shown in patients pertaining not only comprehension skills but also to other language or cognitive abilities subserved by Wernicke's area and surroundings.

Despite these limitations, the present study demonstrated that auditory comprehension transiently improved after a single administration of iTBS over Wernicke's area in patients suffering from fluent aphasia following infarction in perisylvian language areas at chronic stage. Further investigation involving a larger cohort of patients may confirm the effect revealed in the current pilot study and point out whether ipsilesional iTBS on Wernicke's area may be a useful tool for improving language rehabilitation in chronic aphasic patients.

## Data Availability Statement

The datasets generated for this study are available on request to the corresponding author.

## Ethics Statement

The studies involving human participants were reviewed and approved by Ethics Committee Private Medical University Salzburg. The patients/participants provided their written informed consent to participate in this study.

## Author Contributions

VV and RN made substantial contributions to the conception of the work, performed the data interpretation, the drafting/revising of the manuscript, accepted responsibility for conduct of research, and final approval. KS, PL, and SG performed the acquisition and analysis of data, drafting of the manuscript, accepted responsibility for conduct of research, and final approval. LSe, FB, EP-F, and LSa performed the critical revision of the article, accepted responsibility for conduct of research, and final approval.

### Conflict of Interest

The authors declare that the research was conducted in the absence of any commercial or financial relationships that could be construed as a potential conflict of interest.
